# The Importance of Multidisciplinary Management for Placenta Increta: A Case Report

**DOI:** 10.7759/cureus.64242

**Published:** 2024-07-10

**Authors:** Wafa Al Maskeen, Rahaf Almuhaimeed, Nafisah Al Radhwan

**Affiliations:** 1 Obstetrics and Gynecology, Qatif Central Hospital, Qatif, SAU; 2 Obstetrics and Gynecology, Imam Abdulrahman Bin Faisal University, Dammam, SAU

**Keywords:** cesarean section, cesarean hysterectomy, multidisciplinary approach, placental abnormality, placenta increta, placenta previa

## Abstract

This case involves a 34-year-old pregnant woman, gravida 6 para 5, with a gestational age of 32 weeks plus one day. After imaging studies, doctors suspected that she had an abnormal placentation and referred her to a secondary hospital for further management. Surgeons there performed a successful elective cesarean section and a total abdominal hysterectomy with a multidisciplinary approach in mind.

## Introduction

Placenta accreta, placenta increta, and placenta percreta are three different types of abnormal placentation in the placenta accreta spectrum (PAS). Aberrant placental invasion into the myometrium caused by preexisting endometrial-myometrial interface injury characterizes PAS. Placenta percreta (PP) is a condition in which the placenta completely penetrates the myometrium into the serosa and invades the surrounding structures, such as the urinary bladder or rectum, thus complicating the condition and making PP the most fatal in the PAS [1-6]. This is different from placenta accreta when the placenta attaches directly to the muscular lining of the uterus, and from placenta increata where it invades deeply into it [2]. The biggest risk factors of PAS are previous cesarean section and prior history of placenta previa. Other considerations include past myomectomy, uterine curettage, endometrial ablation, uterine abnormalities, and medical operations such as uterine embolization or pelvic irradiation [4]. Significant obstetric bleeding or emergency hysterectomy are the primary causes of maternal morbidity and mortality from PAS [6]. Its efficacy, repeatability, and reduced cost make ultrasound the most common method of evaluating and diagnosing any suspected PAS. Magnetic resonance imaging (MRI) may support ultrasound diagnosis [3]. A multidisciplinary team approach is essential to manage placenta percreta [1].

## Case presentation

Background

**Figure 1:**
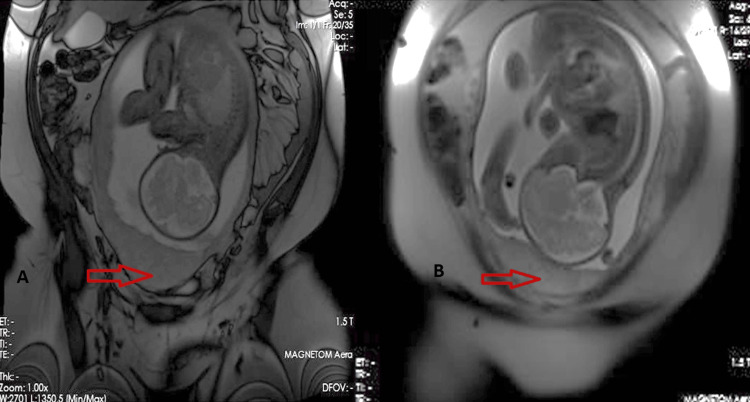
(A) and (B): MRI showing the placenta (red arrows) with dark intraplacental bands

The patient was a 34-year-old pregnant woman, gravida 6 para 5, with a gestational age of 32 weeks plus one day. She had no previous abortions or known chronic medical illnesses. Doctors referred her from Al-Jubail Hospital to Qatif Central Hospital (QCH) as a case of placenta accreta. The patient presented to the Obstetrics and Gynecology Emergency Department complaining of mild lower abdominal pain. Her previous pregnancies were all spontaneous. Para 1 was a full-term normal vaginal delivery, whereas paras 2, 3, 4, and 5 were lower segment cesarean section. Doctors performed a pelvic obstetrics ultrasound upon admission to the Obstetrics and Gynecology ward. The ultrasound showed a single viable fetus with cephalic presentation and placenta posterior covering the cervix and reaching above the previous uterine scar. The myometrium was thin, and a Doppler examination showed vascularity at the anterior uterine wall with fetal parameters corresponding to gestational age calculated from the first day of the last menstrual period. Liquor was adequate, and fetal heart and fetal movement were both positive. Estimated fetal weight was approximately 1.996 grams +− 291 grams. An MRI showed a heterogeneous placenta, dark intraplacental bands, uterine bulging, loss of the retroplacental clear space, and reduced myometrial thickness (Figure 1).

The obstetrics and gynecology team ordered a complete blood count test, biochemistry blood test, liver function test, urine examination, and coagulation profile. All appeared normal. Cross-matching tests were also conducted. The preoperative haemoglobin level was 12.3 g/dL.

Surgical management

**Figure 2:**
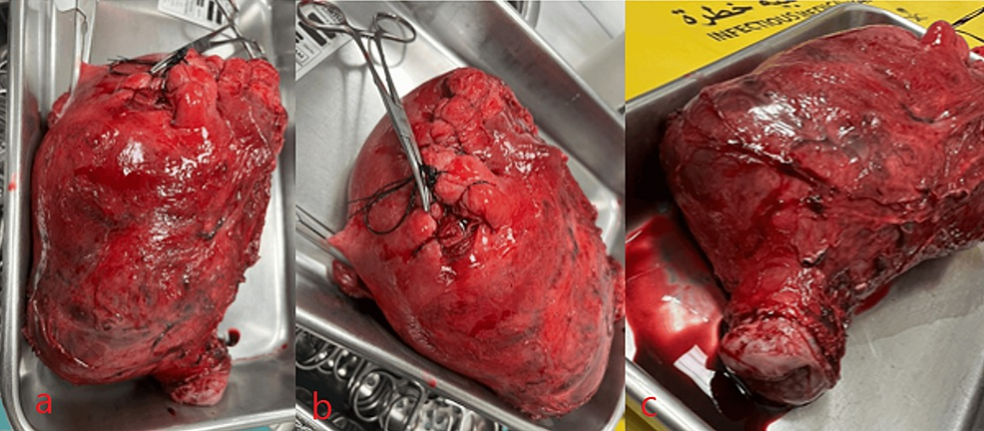
(A), (B), and (C): uterus and cervix

The obstetrics and gynecology team gave the patient dexamethasone at the gestational age of 31 weeks and 6 days with a dosage of a 6 mg ampule twice daily intramuscularly for 2 days. After that, they scheduled the patient for delivery by elective cesarean section with hysterectomy at the 34th week of gestation. A multidisciplinary approach involved teams from obstetrics and gynecology, anesthesiology, urology, vascular, pediatrics and neonatology, interventional radiology, general surgery, hematology, and the blood bank. The urology team placed the patient under general anesthesia, inserted a Foley catheter, and performed a cystoscopy with double-J stenting. The vascular team performed a perioperative endovascular internal iliac artery occlusion. Then a classical cesarean section (CS) was done by incision at the fundus and anterior uterine wall to the left, then the uterine incision was extended bluntly. Examination under anesthesia followed by classical CS revealed placenta percreta infiltrating the lower segment but no bladder invasion. The doctors successfully delivered the breech fetus, kept the placenta inside the uterus, and used no uterotonic medication. They then performed a total abdominal hysterectomy (Figure 2). The estimated blood loss was 800 ml.

Medical personnel took the patient to the ICU in stable condition for close observation for 24 hours and then moved her to the ward to develop postoperative ileus that was found by abdominal X-ray. The patient was managed conservatively by the general surgery department and a nasogastric tube was inserted. The patient’s condition had sufficiently improved by Day 4 after surgery to justify her discharge. The baby was taken to the neonatal intensive care unit after the operation, was handled by the neonatology team, and then was shifted to the nursery shortly after that, as the baby was stable. The baby was discharged home with his mother. The patient’s discharge hemoglobin level was 13.5 g/dL. Doctors prescribed enoxaparin 60 mg subcutaneously once daily for two weeks, paracetamol 1 g orally once daily as needed, calcium 600 mg once daily, and iron and folic acid supplementation orally once daily. Medical personnel sent the uterus (with placenta inside) and the cervix for pathological evaluation. The report suggested placenta increta, thinned myometrium, placenta parenchyma, fetal membranes, and a trivascular umbilical cord with no significant histopathologic abnormalities.

## Discussion

The most generally reported risk factors for PAS are placenta previa, prior uterine surgery (mainly a cesarean section or curettage), and multiparity [1,7]. Our 34-year-old patient presented with an obstetric history of G6P5+0 and four previous cesarean sections. This put her at an increased risk of PAS after each subsequent cesarean section. This is congruent with a recent meta-analysis that screened for placenta accreta among 40,000 pregnancies with prior placenta previa, low-lying placenta, or prior cesarean birth, which showed that the prevalence of placenta previa accreta was 4.1% in women with one prior cesarean delivery and 13.3% in women with two prior cesarean deliveries [8]. PAS can lead to a massive hemorrhage, which is considered a major obstetric emergency, particularly when the diagnosis is missed in the antenatal period, which may necessitate an emergency hysterectomy to control the bleeding [6]. Therefore, developing a prenatal ultrasound screening protocol is critical to assess the prenatal risk of accreta placentation, especially among women with low-lying placenta previa and a history of prior cesarean birth [8]. The presence of dysfunctional vasculature on color Doppler ultrasound had the best combination of sensitivity and specificity for predicting invasive placentation, whereas abnormalities of the uterus-bladder interface had the highest specificity [9].

The American College of Obstetricians and Gynecologists and the Society for Maternal-Fetal Medicine argue that ultrasound intervals of 18-20, 28-30, and 32-34 weeks of gestation in asymptomatic patients could aid in early localization of placental abnormalities [10]. Clinically, painful antepartum bleeding is the most prevalent clinical manifestation of placenta percreta (72%), followed by postpartum hemorrhage (68%). Placental abruption could cause abdominal pain, which could be a warning sign for uterine rupture owing to placenta percreta. Meanwhile, hematuria affects 5% of patients [11].

Although the clinical presentation of our patient was limited to persistent mild to moderate lower abdominal pain, doctors at her primary hospital established the preliminary diagnosis as placenta accreta with speculation of the involvement of the bladder, according to ultrasound and MRI imaging studies. They referred her to QCH for further management. Further postoperative testing, including histopathological studies, confirmed the diagnosis of placenta increta. The histopathological sample taken intraoperatively confirmed the diagnosis and differentiation among the PAS classification.

The diagnostic accuracy of ultrasound and MRI for PAS is comparable. Thus, scholars argue that we should base the choice of initial imaging modality as a screening for suspected PAS on equipment availability and examiner experience [12]. The optimal management of PAS is dependent on early antenatal recognition of the diagnosis. However, limitations in imaging techniques or availability in primary centers or atypical presentation sometimes render such recognition difficult.

Fortunately, in this case, doctors at Al-Jubail Hospital established the preliminary diagnosis via imaging studies. The preoperative conditioning and planning with the multidisciplinary team consisting of gynecologists, urologists, hematologists, anesthesiologists, and vascular surgeons commenced early at QCH. Because an appropriate operative approach may differ case by case [11,12], we held a meeting preoperatively in the Obstetrics Department at QCH to discuss the operative plan. We advised the patient and her husband about the risks of the procedure, possible complications and sequelae of infertility, and possible removal of the bladder if we found profound invasion intraoperatively. After obtaining their consent, we considered the operative options, which ranged from a conservative approach (with cesarean hysterectomy, the placenta left in situ combined with methotrexate chemotherapy, and then a delayed hysterectomy) to a nonconservative approach that included a cesarean hysterectomy [6,13]. In our operating theater, we performed a cesarean hysterectomy to deliver the baby in a breech position leaving the placenta in situ. Following the closure of the uterus, we performed a total abdominal hysterectomy under general anesthesia. The vascular team performed a perioperative endovascular internal iliac artery occlusion followed by the urology team that performed a cystoscopy with double-J stenting of the ureters. The estimated blood loss intraoperatively was around 800 ml. We delivered the baby and sent the resected uterus and cervix to the Pathology Department. The postoperative period is also significant in influencing the outcomes as well as establishing a close follow-up [10]. Complications could range from heavy bleeding, disseminated intravascular coagulation, laceration failure, fistula formation, and ileus [14]. Our patient developed an ileus, which resolved after 24 hours, and an incisional hernia later on. Scholars commonly report these as general postoperative complications [14-16]. Otherwise, we evaded other serious complications.

## Conclusions

Keeping a high index of suspicion is important for the early diagnosis of PAS because early antenatal recognition of abnormal placentation is essential to begin the appropriate management. Atypical clinical presentation and the availability of imaging modalities like MRI and expertise could complicate this. This case illuminates that the right referral to a center with an accessible multidisciplinary team of obstetricians, gynecologists, vascular surgeons, urologists, anesthesiologists, hematologists, and the blood bank with blood transfusion medicine is key for optimizing patient outcomes and establishing good follow-up to manage any potential postoperative complications. In conclusion, evidence of this is found in our case, which was managed successfully due to early diagnosis and referral to a higher facility capable of handling PAS.
